# Diagnostic Accuracy of Coronary Artery Occlusion and Myocardial Perfusion Defect on Non-Gated Enhanced Chest CT in Predicting Acute Myocardial Infarction

**DOI:** 10.3390/tomography7040043

**Published:** 2021-09-27

**Authors:** Min Ji Son, Dongjun Lee, Seung Min Yoo, Charles S. White

**Affiliations:** 1 Department of Radiology, CHA University Bundang Medical Center, Seongnam 13497, Korea; smj3006@chamc.co.kr (M.J.S.); sirious0416@chamc.co.kr (D.L.); 2 Department of Radiology, University of Maryland, Baltimore, MD 20742, USA; cwhite@umm.edu

**Keywords:** acute myocardial infarction, coronary artery occlusion, myocardial perfusion defect, chest CT, CT

## Abstract

The purpose of this study was to evaluate the diagnostic accuracy of coronary artery occlusion (CAO) and myocardial perfusion defect (MPD) identified on non-gated enhanced chest CT in patients with acute myocardial infarction (AMI). We retrospectively assessed 99 patients with AMI (group 1, *n* = 33) and without AMI (group 2, *n* = 66) who underwent non-gated chest CT. We analyzed the presence of MPD and CAO on non-gated chest CT. MPD on the CT was categorized using a three-point scale (0 = no definite MPD; 1 = probable artifact or questionable MPD; 2 = probable MPD). Presence of CAO was defined as an abrupt change of contrast enhancement in a coronary artery segment with no or minimal coronary motion on the CT. There were 42.4% and 12.1% patients with probable MPD (*p* = 0.002), and 18.2% and 0% patients with CAO (*p* = 0.001) in groups 1 and 2, respectively. Probable MPD alone and simultaneous presence of CAO and probable MPD to predict AMI resulted in sensitivity, specificity, negative predictive value, and positive predictive valve of 42.4%, 87.9%, 75.3%, and 63.6%, respectively, and 12.1%, 100%, 69.5%, and 100%, respectively. In conclusion, probable MPD alone on non-gated chest CT demonstrated a relatively low sensitivity, high specificity, and modest positive predictive value for the prediction of AMI on non-gated enhanced chest CT. Although it is rare, simultaneous presence of CAO and probable MPD had a high positive predictive value to predict AMI on non-gated enhanced chest CT.

## 1. Introduction

Non-gated enhanced chest CT can be a first-line imaging study in patients who present with nonspecific chest pain, shock, or dyspnea, and who prove to have acute myocardial infarction (AMI) [1]. This situation may occur particularly in the emergency department, where highly sensitive troponin testing and 24 h coverage with ECG-gated coronary CT angiography (CTA) is not available. On non-gated enhanced chest CT, the finding of a definite myocardial perfusion defect (MPD) is an important diagnostic clue to the presence of AMI, independent of an increase in cardiac specific enzymes or ischemic ECG changes [2,3,4].

However, the clinical impact of this CT sign may be limited by the presence of a false positive or negative diagnosis, mainly due to motion or beam-hardening artifacts on non-gated enhanced CT [4]. Similarly, noninvasive evaluation of coronary arteries is typically performed in ECG-gated coronary CTA, not in non-gated enhanced chest CT due to frequent coronary motion blurring [5,6,7,8].

However, the presence of a wall motion abnormality leading to reduced motion of the epicardial coronary artery in the culprit arterial territory in patients with AMI may increase the opportunity to identify coronary artery occlusion (CAO), even on non-gated enhanced chest CT. In this context, it may be valuable to precisely discriminate true MPD and CAO from the artifacts on the non-gated enhanced chest CT in patients with AMI, while minimizing false positive diagnosis by applying strict criteria on probable MPD and CAO. Therefore, the purpose of this study was to investigate whether presence of probable MPD or CAO with least motion blurring on the non-gated enhanced chest CT have a high specificity to predict AMI or not.

## 2. Materials and Methods

### 2.1. Study Population

Approval of the CHA University Bundang Medical ceneter, the institutional review board (protocol number: 2016-06-016) was obtained, and informed consent was waived for this study. Between September 2010 and December 2017, using a radiological and clinical database at a university hospital, we identified 45 patients who underwent non-gated enhanced chest CT for a nonspecific clinical presentation within 72 h prior to diagnosis of AMI. Non-gated chest CT instead of coronary CTA was indicated in the enrolled patients due to low clinical pretest probability of AMI or unavailability of coronary CTA during the night. The diagnosis of AMI was confirmed using criteria of the third universal definition of AMI [9]. We excluded patients for the following reasons (chronic myocardial infarction (*n* = 2), previous coronary stent insertion (*n* = 3), poor myocardial enhancement (*n* = 3), no coronary angiography (*n* = 4)). Thus, our final cohort consisted of 33 patients with AMI (group 1) who underwent non-ECG gated enhanced chest CT prior to diagnosis in this study. We also assessed a group of 66 randomly selected age, and sex-matched patients without a final diagnosis of AMI (group 2) who underwent non-gated chest CT for a non-specific clinical presentation as a control group.

We analyzed the following clinical and laboratory findings: age, sex, body mass index (BMI, kg/m^2^), hypertension, diabetes mellitus, smoking, systolic and diastolic blood pressure, blood glucose, triglyceride, total cholesterol, high-density lipoprotein levels, low-density lipoprotein levels, and C-reactive protein level. Hypertension was defined as systolic blood pressure >140 mmHg and diastolic blood pressure of >90 mmHg or treatment with antihypertensive medications. Diabetes mellitus was defined as fasting blood glucose level (≥126 mg/dL) or treatment with anti-diabetic medications. We also analyzed the chief complaint of the patient and the initial radiologic report to assess whether there was a false negative diagnosis.

### 2.2. CT Technique

Non-ECG gated enhanced chest CT was performed on a 64-slice multi-detector CT (LightSpeed VCT, GE HealthCare, Milwaukee, WI, USA). Scanning parameters were as follows: 100 or 120 kV, 100–400 mA, 500 ms gantry rotation time, 64 × 0.625 mm detector collimation, 0.625 *z*-axis resolution, 1 pitch, and 3 mm reconstruction thickness. A total of 60 to 100 mL of intravenous Ioversol (Optiray 320 mg/mL, Tyco Healthcare, Montreal, QC, Canada) was injected at a flow rate of 4–5 mL/s based on BMI. Non-gated chest CT was performed after 60–75 s after administration of contrast media.

### 2.3. Image Analysis

**Figure 1:**
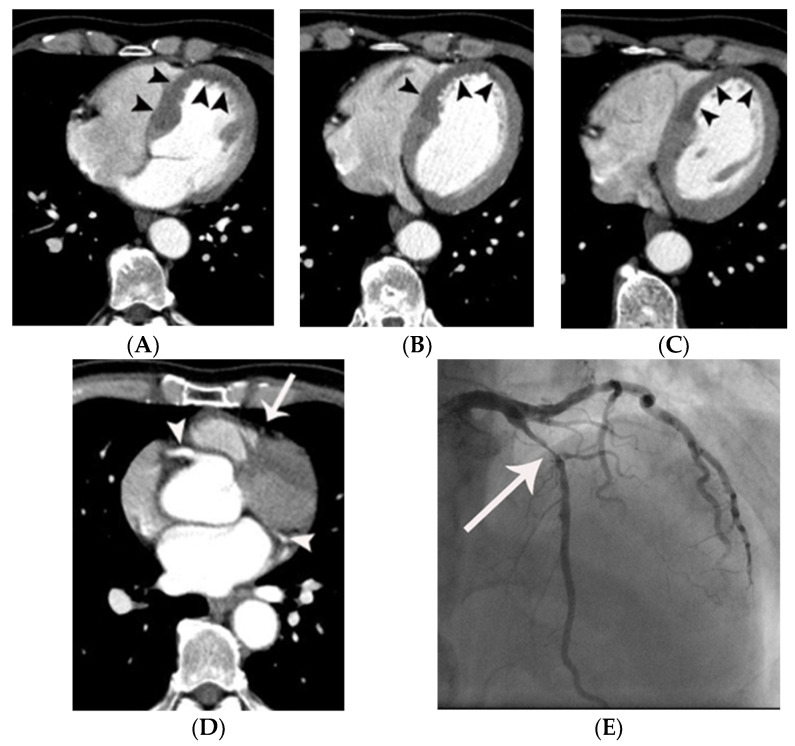
Representative case, demonstrating probable MPD in a 69-year-old male with acute chest pain. Subendocardial or transmural hypoattenuation (arrowheads in (**A**–**C**)) was demonstrated on the multiple non-gated axial CT images along the left anterior descending coronary territory (i.e., antero-septum and the left ventricular apex). Note that attenuation of the mid-left anterior descending coronary artery (arrow in (**D**)) is low compared to the right coronary artery and left circumflex coronary artery (arrowheads in (**D**)), suggesting critical narrowing or occlusion of the left anterior descending coronary artery. Critical narrowing in the proximal left anterior descending coronary artery was confirmed on subsequent coronary angiography (arrow in (**E**)).

**Figure 2:**
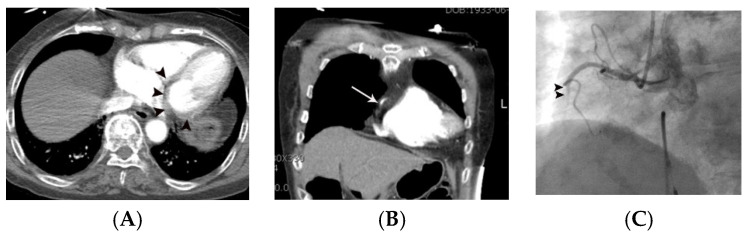
Coronary artery occlusion in the mid-right coronary artery demonstrated on non-gated enhanced chest CT in a 67-year-old woman with acute myocardial infarction. (**A**) Artifact vs. true myocardial perfusion defect (arrowheads) was noted in the inferior wall of the left ventricle on an axial image. Suspicion of acute myocardial infarction was not described in the initial radiologic report. (**B**) On retrospective analysis of the coronary arteries, an abrupt cut-off (arrow) of contrast was noted in the mid-right coronary artery on a coronal reformatted image. Note that only minimal motion artifact is present in this image, even though the image was obtained without ECG gating. (**C**) Subsequent emergent coronary angiography confirmed the non-gated chest CT finding (arrowheads).

A retrospective evaluation was performed by two radiologists with 16 and 5 years of experience, respectively, in a consensus manner for CT grading for MPD vs. artifact on non-ECG gated enhanced chest CT. Reviewers were blinded to the initial CT report and the clinical, echocardiographic, and invasive angiographic findings. Myocardial enhancement on the CT was categorized using a three-point scale (0–2) as follows: CT grade 0 = no defect; CT grade 1 = probable artifact or questionable MPD not fully satisfying CT grade 2, mainly due to image degradation by motion; CT grade 2 = probable MPD following a vascular territory with minimal motion artifact (i.e., MPD in the multiple CT levels along the corresponding arterial territory) (Figure 1) [2]. In this study, probable artifact and questionable MPD was classified as the same score for MPD, because the differentiation between the two is often difficult. In order to minimize the false positive rate, we considered a CT score of 2 for MPD to be positive for the diagnosis of AMI. We also analyzed presence of myocardial thinning in a territory showing MPD. Myocardial thinning was defined as the presence of myocardial segment or segments in the left ventricle with disproportionally thinner myocardial thickness compared with the remaining left ventricular wall on CT. Four weeks after grading the CT studies for possible MPD, an independent session was held to evaluate coronary artery occlusion. CAO in a coronary segment was deemed to be present in the setting when satisfying all of the following CT findings: (1) an abrupt change of contrast enhancement suggesting occlusion by mainly non-calcified plaque; (2) no or only minimal coronary motion; and (3) absence of negative remodeling (i.e., smaller diameter compared to that of adjacent normal coronary segment). We included the third condition in the definition of CAO because most of the culprit plaques resulting in AMI are associated with positive remodeling, not negative remodeling [1]. We excluded cases not fulfilling the above criteria, often by coronary motion or blooming artifact. CT attenuation was measured at the lowest attenuation (size of region of interest = 5 mm^2^) within the CAO. The vessel site with CAO on non-gated chest CT was required to be in the same location as the culprit lesion on subsequent coronary angiography (Figure 2). The presence of a wall motion abnormality on echocardiography and location of the culprit plaque on coronary angiography were analyzed by a cardiologist blinded to CT findings.

### 2.4. Statistical Analysis

We analyzed whether there was a significant difference in the CT findings between the two groups. Statistical analysis (SPSS Inc., Chicago, IL, USA) was performed using Fisher’s exact test and Student’s *t*-test for categorical and continuous variables, respectively. A statistically significant difference was defined as *p* < 0.05.

## 3. Results

### 3.1. Patient Characteristics

There were no significant differences in the demographic and clinical characteristics potentially related to AMI between the two groups. Table 1 summarizes the demographic and clinical characteristics of the patient cohort.

### 3.2. Incremental Value of CAO in the Diagnosis of AMI over Evaluation of MPD Alone

The frequency of false negative diagnosis of AMI on the initial CT report in patients who presented with acute chest pain (*n* = 21) as compared to other presentations (*n =* 12; dyspnea, *n* = 10; shock, *n* = 2) was 42.9% (9/21) and 91.6% (11/12), respectively (*p* = 0.02). There was no case showing myocardial thinning.

**Figure 3:**
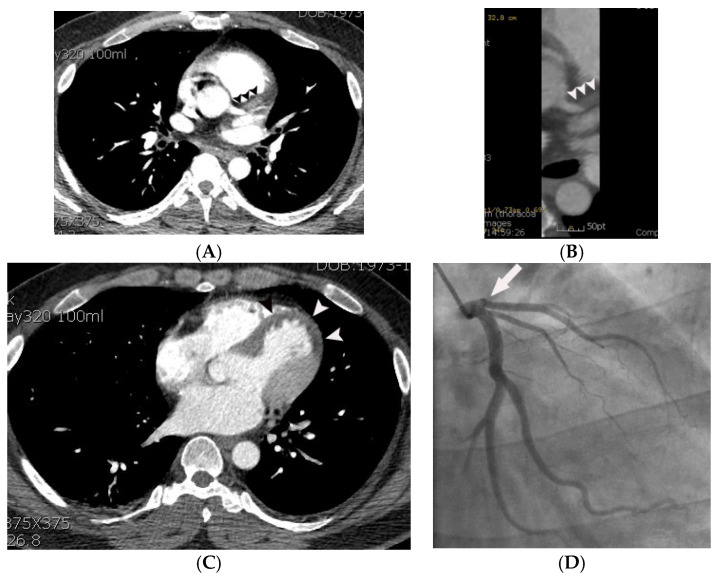
Coronary artery occlusion demonstrated on non-gated enhanced chest CT in a 44-year-old man with acute myocardial infarction. (**A**) An abrupt cut-off (arrowheads) of contrast was noted at the origin of the left anterior descending coronary artery on an axial image at the level of the aortic root. Note that only minimal motion artifact is present in this non ECG-gated image. (**B**) The same finding (arrowheads) was demonstrated more clearly on a curved multi-planar reformatted image. (**C**) A probable myocardial perfusion defect (CT grade 2) (arrowheads) was noted in the left ventricular apex on an axial image at the level of the left atrium. (**D**) Subsequent coronary angiography confirmed the CT finding (arrow).

There were significant differences in the prevalence of normal, probable artifact or equivocal MPD, and probable MPD between the two groups (Table 2). There were 18.2% (6/33) and 0% (0/66) of patients showing CAO on non-gated chest CT in groups 1 and 2, respectively (*p* = 0.001). The location of the culprit lesion in the coronary arteries was variable in group 1 (Table 3) (Figure 2 and Figure 3).

**Figure 4:**
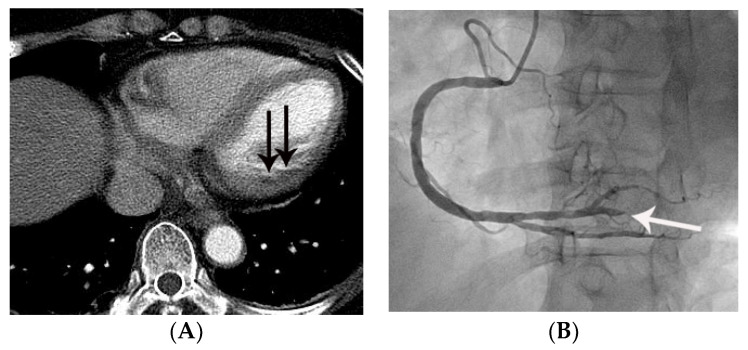
False negative diagnosis of acute myocardial infarction on non-gated chest CT in a 67-year-old female with a small acute myocardial infarction. (**A**) Equivocal MPD (CT score of 1 for myocardial perfusion defect (MPD)) (arrow) was noted in the basal lateral segment of the left ventricle on an axial image. (**B**) Subsequent coronary angiography showed total occlusion (arrow) of the posterolateral branch of the right coronary artery, suggesting a culprit lesion. Note that it was difficult to differentiate true MPD from artifacts in this case.

**Figure 5:**
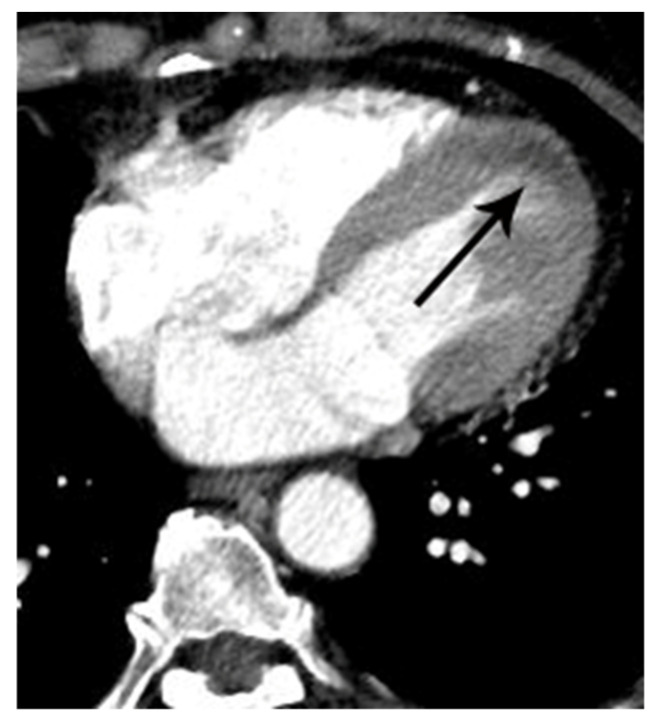
False positive myocardial perfusion defect (MPD) in a 55-year-old female with acute chest pain. Focal low attenuation (arrow) in the left ventricular apex was noted on an axial CT image. The lesion was given a CT score of 2 for MPD. However, serial ECG and serum cardiac troponin were normal, and her final diagnosis was reflux esophagitis.

There were no cases of left circumflex coronary artery (LCX) occlusion on CT, although there were two cases of LCX occlusion on coronary angiography. The mean value of CT attenuation at the CAO was 59.6 ± 22.0 Hounsfield unit (HU) (range: 37–87). In all the enrolled patients (*n* = 99), there was at least one coronary segment demonstrating severe coronary motion (*n* = 99) or blooming artifact (*n* = 67) in the present study. There was no case demonstrating CAO with negative remodeling in this study. Wall motion abnormality by echocardiography was noted in 78.8% (26/33) of the AMI patients. More specifically, wall motion abnormality in the corresponding arterial territory was demonstrated in all patients who showed CT evidence of CAO (100%, 6/6) (Table 4). In this study, a cut-off value of MPD ≥ 1 to predict AMI resulted in high sensitivity (87.9%, 29/33), but relatively low to modest specificity (63.6%, 42/66) (Table 5). In contrast, probable MPD alone and simultaneous presence of CAO and probable MPD to predict AMI resulted in sensitivity, specificity, negative predictive value, and positive predictive value of 42.4%, 87.9%, 75.3%, and 63.6%, respectively, and 12.1%, 100%, 69.5%, and 100%, respectively (Table 5) (Figure 4 and Figure 5).

## 4. Discussion

The key finding of this study was identification of clinical and radiological factors related to a false negative diagnosis of AMI on non-gated enhanced chest CT. These include presentation with symptoms other than acute chest pain and a low CT score for MPD (i.e., inability to distinguish a true perfusion defect from artifact). Thus, when interpreting non-gated chest CT, attention should be paid to both the myocardium and coronary arteries, particularly in patients who present with dyspnea or shock. The presence of focal lower CT attenuation within the left ventricular myocardium indicates two possibilities (artifact and true MPD). In cases demonstrating equivocal MPD (CT score of 1 for MPD) on non-gated chest CT, it was difficult to precisely discriminate artifacts from true MPD. Thus, we only included probable MPD (CT score of 2 for MPD) as a cutoff value for prediction of AMI in this study. As the use of probable MPD increased the specificity to predict AMI, this was associated with decreased sensitivity in this study (Table 4). Notably, it was assumed that a small AMI was more likely to be associated with a false negative result on MPD on CT (Figure 4) compared to AMI involving a larger area of the left ventricle (Figure 3). In addition, the ability to differentiate true MPD from artifacts may be different depending on the experience of the interpreting radiologist. Thus, we recommend that further testing with tools such as cardiac troponins and ECG be performed in patients showing equivocal MPD on non-gated chest CT to differentiate AMI from the artifact.

The other key finding of the current study was that simultaneous presence of CAO and probable MPD on non-gated enhanced chest CT may be an important clue in the diagnosis of AMI due to its high positive predictive value, although it is a rare occasion. In fact, presence of CAO alone on non-gated chest CT was also 100% specific for the diagnosis of AMI in this study. However, the result was not validated in the study with a large number of the enrolled patients. Thus, differentiation from true CAO from the false positive CAO by motion artifact may be difficult on non-gated chest CT, especially in patients with small equivocal MPD (i.e., isolated CAO). Further studies are recommended to address this issue.

Notably, chronic total occlusion (not included in this study) predominantly consisting of non-calcified plaque may have similar CT findings with acute CAO on non-gated chest CT. In addition, cases with chronic total occlusion with old myocardial infarction can also present CAO and MPD. However, old myocardial infarction on non-gated chest CT can often be differentiated from AMI with presence of myocardial thinning or fatty metamorphosis. In the present study, there was no case showing myocardial thinning. We only assumed that the subacute phase of AMI may demonstrate similar CT findings on non-gated chest CT to those of typical AMI. In this occasion, presence of acute clinical presentation, elevation of serum troponin, and ischemic ECG change may favor the possibility of AMI rather than subacute AMI. In clinical practice, working diagnosis of AMI on non-gated chest CT is possible even before the elevation of serum troponin. Thus, this may have a potential to reduce door-to-balloon time. To date, there has been only one case report that reported CAO on non-gated enhanced chest CT [10]. However, identification of CAO on the non-gated chest CT was not extremely rare in this study, in contrast to scarcity of the relevant reports in the English literature. In this study, simultaneous presence of CAO and probable MPD showed low sensitivity (12.1%, 4/33), but 100% specificity (66/66). Importantly, there may be still possibility for false positive diagnosis of AMI when radiologists encounter a MPD (CT score of 2 for MPD) on non-gated enhanced chest CT (Figure 5). Thus, identification of such a combination may increase diagnostic confidence of AMI on non-gated enhanced chest CT (i.e., increased positive predictive value compared to probable MPD alone).

Possible explanations for the ability to visualize CAO on non-gated enhanced chest CT include the following. First, the epicardial portion of the coronary artery lies in close proximity to the left or right ventricle. Thus, akinesia or hypokinesia of the ventricle in patients with AMI tends to make the epicardial coronary artery less mobile and more visible. Second, features of the plaque may assist in identification. In one recent study using dedicated coronary CTA, many culprit lesions in patients with AMI were characterized by CAO consisting of substantial thrombosis and marked positive remodeling. In contrast, thrombus in culprit plaques could not be differentiated from non-calcified plaque based solely on CT HU; thus, bulky nature of the culprit plaques may permit identification of CAO on enhanced chest CT even if gating is not used [11]. Using the high-pitch mode of dual-source CT with shorter temporal resolution, the frequency of detection of CAO on non-gated enhanced CT may be higher than in our study, which used single-source CT, although this would need to be confirmed in a future study [12].

This study was limited by its small retrospectively selected cohort, which may have led to selection bias. A follow-up prospective study with a large number of patients would be ideal. The second, the scan time to begin the enhanced chest CT in this study (60–75 s) was different from the earlier CT (100 s). This may have resulted in a difference in the diagnostic accuracy for MPD compared to prior studies [4,13]. Third, the authors used a visual CT score for classification of MPD rather than HU difference, as was used in this study. This was done for the following reasons: (1) accurate measurement of CT attenuation may be difficult on non-gated chest CT due to motion or beam hardening artifact; and (2) visual assessment seems to be a more suitable way to assess the myocardium in clinical practice.

## 5. Conclusions

Probable MPD alone on non-gated chest CT demonstrated a relatively low sensitivity, high specificity, and modest positive predictive value for the prediction of AMI on non-gated enhanced chest CT. Although it was rare, simultaneous presence of CAO and probable MPD had a high positive predictive value to predict AMI on non-gated enhanced chest CT.

## Figures and Tables

**Table 1:** The demographic and clinical characteristics of the patient cohort.

Variables	Means ± SD or Prevalence		
	Group 1	Group 2	*p* Value
Age (years)	62 ± 14.1	63 ± 25.2	>0.05
Sex (male/female)	25/8	49/17	>0.05
Body mass index (kg/m^2^)	23.9 ± 3.7	23.7 ± 3.0	>0.05
Hypertension	22/33 (66.7)	39/66 (59.1)	>0.05
Systolic blood pressure (mmHg)	133.2 ± 17.9	132.2 ± 16.8	>0.05
Diastolic blood pressure (mmHg)	77.9 ± 80.1	78.9 ± 78.1	>0.05
Smoking	20/33 (60.6)	36/66 (54.5)	>0.05
Diabetes mellitus	14/33 (42.4)	23/66 (34.8)	>0.05
High density lipoprotein (mg/dL)	41.0 ± 9.8	41.7 ± 8.5	>0.05
Low density lipoprotein (mg/dL)	105.6 ± 33.6	108.6 ± 41.4	>0.05
C-reactive protein (mg/dL)	2.2 ± 5.1	2.1 ± 5.1	>0.05

SD indicates standard deviation. Parentheses indicate percentage.

**Table 2:** Differences in the CT scores for myocardial perfusion defect between groups 1 and 2.

	Group 1	Group 2	*p* Value
No defect (CT score of 0)	4/33 (12.1)	42/66 (63.6)	0.0001
Probable artifact or equivocal MPD (CT score of 1)	15/33 (45.5)	16/66 (24.2)	0.04
Probable MPD (CT score of 2)	14/33 (42.4)	8/66 (12.1)	0.002

Parentheses indicate percentage. MPD indicates myocardial perfusion defect.

**Table 3:** Location of culprit lesion on coronary angiography.

Left main artery	1
Proximal left anterior descending artery	9
Middle left anterior descending artery	14
First diagonal branch	1
Proximal right coronary artery	1
Middle right coronary artery	3
Distal right coronary artery	1
Posterolateral branch	1
Proximal left circumflex artery	1
Distal left circumflex artery	1

**Table 4:** Association between wall motion abnormality on echocardiography and myocardial perfusion defect (MPD) on non-gated enhanced chest CT in group 1.

	Positive Wall Motion Abnormality	No Wall Motion Abnormality
No defect (CT score of 0)	0/33 (0)	4/33 (12.1)
Probable artifact or equivocal MPD (CT score of 1)	12/33 (36.4)	3/33 (9.1)
Probable MPD (CT score of 2)	14/33 (42.4)	0/33 (0)
Coronary artery occlusion	6/6 (100)	0/33 (0)

Parentheses indicate percentage. MPD indicates myocardial perfusion defect.

**Table 5:** Accuracy of the diagnosis of acute myocardial infarction on non-ECG gated chest CT according to retrospective CT grading of myocardial perfusion defect or artifact, with addition of coronary artery occlusion.

Variables	Sensitivity	Specificity	Negative Predictive Value	Positive Predictive Value
MPD ≥ 1	29/33 (87.9)	42/66 (63.6)	42/46 (91.3)	29/53 (54.7)
MPD ≥ 2	14/33 (42.4)	58/66 (87.9)	58/77 (75.3)	14/22 (63.6)
CAO alone	6/33 (18.2)	66/66 (100)	66/93 (71)	6/6 (100)
MPD ≥ 2 and CAO	4/33 (12.1)	66/66 (100)	66/95 (69.5)	4/4 (100)

Parentheses indicate percentage. MPD and CAO indicate myocardial perfusion defect and coronary artery occlusion, respectively.

## Data Availability

Not Applicable.
